# In Darkest Africa

**Published:** 1908-02

**Authors:** 


### EDITORIAL DEPARTMENT.

### IN DARKEST AFRICA.

We have received the first report of the Wellcome Research Laboratories at the Gordon Memorial College, Khartoum, Sudan, Africa. The director is Andrew Balfour, M. D., B. Sc., M. R. C. P., Edin.; D. P. H., Camb.; Fellow Royal Institute of Public Health; member of the Epidemiological Society; Medical Officer of Health, Khartoum, and Sanitary Adviser to the Sudan Civil Medical Department, Department of Education, Sudan government, Khartoum, 1904.

This report is intensely interesting. It is in the main a study of several species of mosquito and other pest-breeding insects in that sub-tropical country, and the methods employed in destroying them. The mosquitoes in Khartoum and Omdurman have been completely destroyed. Think of it! Exterminating the mosquitoes on the shores of the Blue Nile, where less than two decades ago the fanatical savages of the Mahdi exterminated the English army—massacred them at Khartoum, including their general, the famous "Chinese" Gordon! The English government has established a great memorial college on the spot, and an American citizen—now a Londoner—Mr. Henry S. Wellcome, the famous chemist, endowed the Research Laboratory. The work that it has done is of inestimable value, even if it were confined to the extermination of the mosquito alone, for thereby malaria, dengue and filariasis (of which elephantiasis is a type) are disarmed and prevented. Dr. Balfour says the *stegomyia* is rare, and as yellow fever is unknown there this species is not much in the count.

The malaria mosquito there is *pyretophorus costalis,* a species of the anophelina, and it has been found to be both a filaria and a malaria carrier; but the true filaria mosquito—the kind that produces elephantiasis (and that disease is common in Sudan)—is a culex; *c. fatigans.* "Further," says Dr. Balfour, "the recent laborious researches of Dr. Graham, of Beyrout" (*Journal Tropic Med.,* January 2, 1903), "seem to have completely proved that this mosquito is the transgressor in the case of dengue, * * * forming the intermediate host for the protozoon of that disease."

The report is beautifully printed and bound, and is illustrated with colored pictures of a dozen or more species of mosquito, of beetles and flies, and is altogether a most valuable contribution to the interesting study of tropical diseases.

The second report was received long before the first, and was reviewed something like a year or more ago.

The Gordon Memorial College is under the patronage of H. M. the King of England; and the Right Hon. Lord Kitchener,

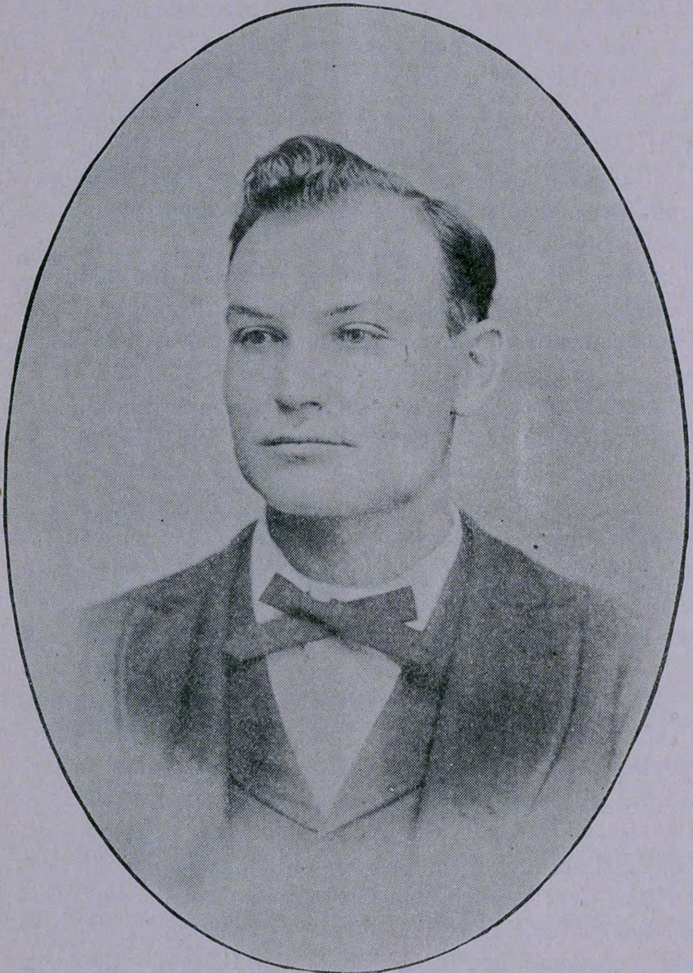

DR. J. D. LAW.

of Khartoum, G. C. B. K. C. M. S., etc., is President. A long list of Right Honorables, including the Right Hon. Sir Ernest Cassell, K. C. M. S., who donated a million dollars to the consumption sanitarium founded by the King.

